# Probing of carotenoid-tryptophan hydrogen bonding dynamics in the single-tryptophan photoactive Orange Carotenoid Protein

**DOI:** 10.1038/s41598-020-68463-8

**Published:** 2020-07-16

**Authors:** Eugene G. Maksimov, Elena A. Protasova, Georgy V. Tsoraev, Igor A. Yaroshevich, Anton I. Maydykovskiy, Evgeny A. Shirshin, Timofey S. Gostev, Alexander Jelzow, Marcus Moldenhauer, Yury B. Slonimskiy, Nikolai N. Sluchanko, Thomas Friedrich

**Affiliations:** 1 Department of Biophysics, Faculty of Biology, Lomonosov Moscow State University, 119991 Moscow, Russia; 2 A.N. Bach Institute of Biochemistry, Federal Research Center of Biotechnology of the Russian Academy of Sciences, 119071 Moscow, Russia; 3 Department of Quantum Electronics, Faculty of Physics, M.V. Lomonosov Moscow State University, 119992 Moscow, Russia; 4 Technical University of Berlin, Institute of Chemistry PC 14, Straße des des 17. Juni 135, 10623 Berlin, Germany; 5 Becker & Hickl GmbH, Nunsdorfer Ring 7-9, 12277 Berlin, Germany

**Keywords:** Biophysics, Biological fluorescence, Molecular biophysics, Biophysical chemistry, Proteins

## Abstract

The photoactive Orange Carotenoid Protein (OCP) plays a key role in cyanobacterial photoprotection. In OCP, a single non-covalently bound keto-carotenoid molecule acts as a light intensity sensor, while the protein is responsible for forming molecular contacts with the light-harvesting antenna, the fluorescence of which is quenched by OCP. Activation of this physiological interaction requires signal transduction from the photoexcited carotenoid to the protein matrix. Recent works revealed an asynchrony between conformational transitions of the carotenoid and the protein. Intrinsic tryptophan (Trp) fluorescence has provided valuable information about the protein part of OCP during its photocycle. However, wild-type OCP contains five Trp residues, which makes extraction of site-specific information impossible. In this work, we overcame this problem by characterizing the photocycle of a fully photoactive OCP variant (OCP-3FH) with only the most critical tryptophan residue (Trp-288) in place. Trp-288 is of special interest because it forms a hydrogen bond to the carotenoid’s keto-oxygen to keep OCP in its dark-adapted state. Using femtosecond pump-probe fluorescence spectroscopy we analyzed the photocycle of OCP-3FH and determined the formation rate of the very first intermediate suggesting that generation of the recently discovered S* state of the carotenoid in OCP precedes the breakage of the hydrogen bonds. Therefore, following Trp fluorescence of the unique photoactive OCP-3FH variant, we identified the rate of the H-bond breakage and provided novel insights into early events accompanying photoactivation of wild-type OCP.

## Introduction

The regulation of excitation energy flows in photosynthetic organisms plays a crucial role for improving biomass production in changing environmental conditions^1^. Carotenoids as essential photosynthetic pigments can contribute to light-harvesting or act as excitation quenchers depending on the structural organization of antennas and the carotenoid state^2^, resulting in excitation energy transfer (EET) to or from chlorophylls, respectively. EET can be conducted by several mechanisms: transfer to (or from) the carotenoid S_1_ excited level^3^, exciton coupling^4,5^ and charge transfer^6,7^. Although functional roles of carotenoids in light-harvesting antennas are clear, there are still debates considering the involvement of specific EET mechanisms in these roles. The situation is additionally complicated by non-Condon nature of the pigment interaction with regard to wave-like EET process and the occurrence of excitation energy coherence^8,9^. Regulation of light-harvesting depends on pH^10^, carotenoid content^11^, lipid content^12^, protein–protein interactions^13^, and other factors^14^, thus highlighting the central role of the protein–pigment complex conformation in energy flow tuning. In higher plants and algae, carotenoid-dependent regulation of light-harvesting can take place directly in membrane-bound light-harvesting complexes (LHCs), as well as in the photosystem II core and minor antennas^15^. However, cyanobacteria represent a very special case by involving photoconvertible water-soluble carotenoproteins.

**Figure 1:**
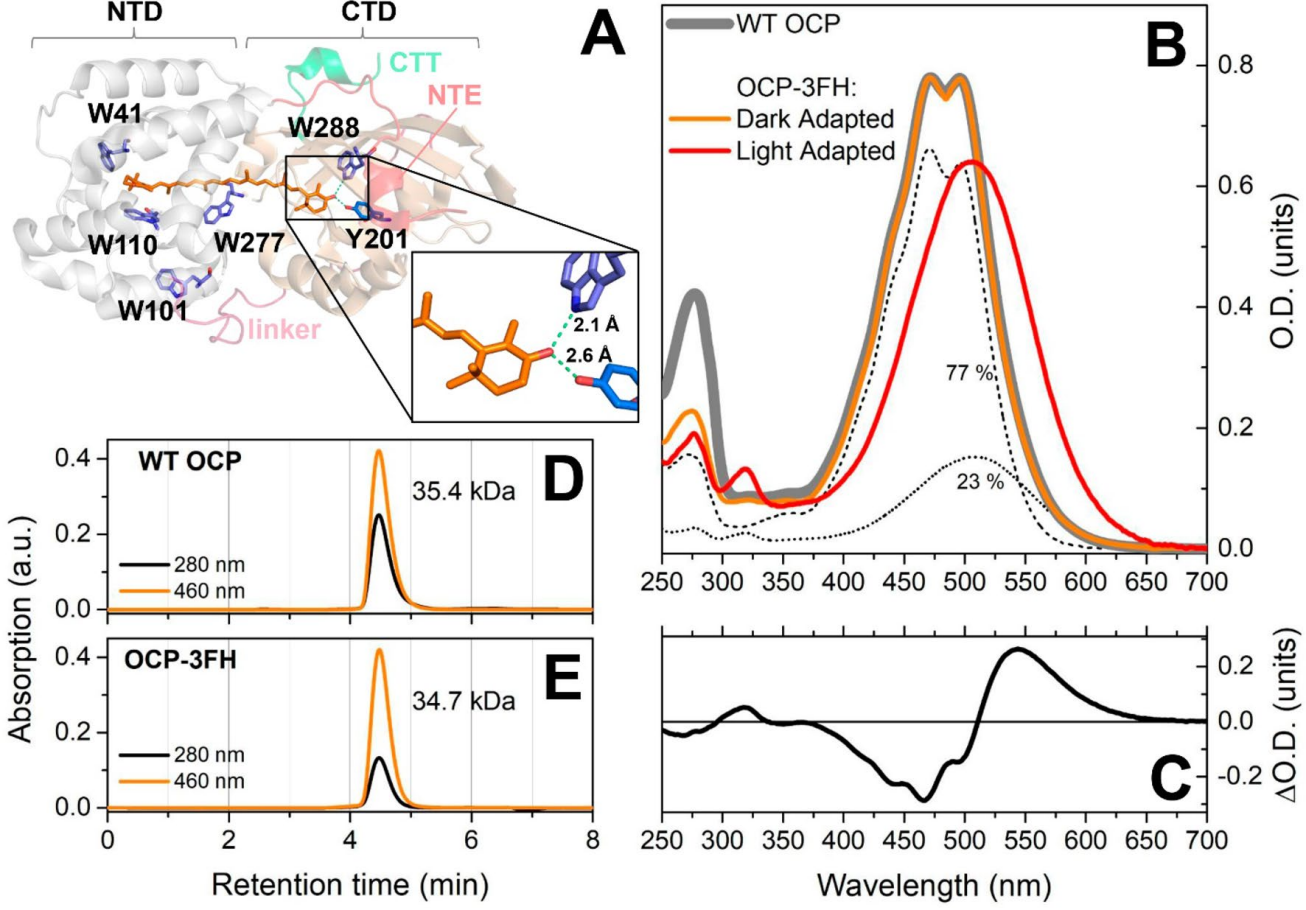
(**A**) Position of Trp residues within the structure of OCP from *Synechocystis *sp. PCC 6803. Trp residues are labeled and shown by violet sticks, the carotenoid-coordinating Tyr-201 is shown by blue sticks, the NTE is shown by red color, CTT is shown by green, the NTD and CTD are shown by grey and beige color, respectively, the interdomain linker is shown in pink. (**B**) The UV–Vis absorption spectra of WT OCP (grey) and OCP-3FH (orange) recorded using a diode array detector in the course of the chromatography run and shown normalized at a position corresponding to the peak maximum (retention time 4.5 min). In order to estimate the yield of distinct spectral states (dotted and dashed lines), the absorption spectrum of the photoactivated protein was subtracted from the dark-adapted sample with an appropriate scaling factor, the numbers indicate corresponding yields. This procedure of spectral decomposition shows that ECN absorption in the compact red state and the final photoconverted state are identical. (**C**) Absorption difference spectrum—photoactivated (red) minus dark-adapted sample (orange). See Supplementary Figs. S1 and S2 for information about the temperature dependency of the OCP-3FH photocycle. Analytical SESC profiles of WT OCP (**D**) and OCP-3FH (**E**) variants followed by 280 nm and 460 nm absorbance, with the Mw estimates obtained from column calibration (see “Materials and methods” for details). In SESC experiments, protein concentration was about 50 µM.

Cyanobacteria perform additional light-harvesting by employing specialized antennas, phycobilisomes, the electronic excitation energy of which eventually reaches the chlorophylls in the photosynthetic reaction centers (RCs), altogether increasing the RCs’ effective absorption cross-section and, thus, the efficiency of oxygenic photosynthesis^16–18^. However, under high levels of insolation, excited states of antenna pigments might cause the formation of reactive oxygen species (ROS)^19^. In order to prevent ROS formation, cyanobacteria uncouple excitation energy transfer from phycobilisomes to reaction center chlorophylls by recruiting a single keto-carotenoid molecule to the phycobilisome core. Carotenoids are characterized by short lifetimes of excited states^20,21^; thus, they can accept energy from the antenna and rapidly convert it into heat^22^. As the carotenoid molecule is insoluble in water and cannot discriminate between normal and high light conditions, a special 35 kDa water-soluble protein is required to modulate its quenching abilities and to deliver the quencher to the phycobilisome core in order to control primary photosynthetic processes. This pigment–protein complex is called the Orange Carotenoid Protein (OCP)^23–27^. It has a modular structure^28^ with different functional activities of its two structural domains^29–33^. The N-terminal domain (NTD) is responsible for the interaction with the phycobilisome, while the C-terminal domain (CTD) is necessary to keep OCP inactive when photoprotection is not required (Fig. 1A). A few contacts between the protein domains exist, as well as hydrogen bonds between the carotenoid’s keto-oxygen and two important residues, tyrosine-201 and tryptophan-288, together stabilizing OCP in its inactive dark state^34^, which is characterized by its orange color in solution. Simultaneously, the protein creates a specific environment for the keto-carotenoid, making it sensitive to light and photoactive^35–37^. However, the photoconversion of OCP is extremely inefficient as the quantum yield for formation of the red, physiologically active form of OCP is approximately 0.2% under short saturating flashes of light^38^. Together with the extremely long lifetime of the activated OCP state(s) (~ 10 s), this makes it difficult to study elementary stages of the OCP photocycle by any spectroscopic approach.

Recent publications showed that the photocycle of OCP proceeds via multiple intermediate states preceding the formation of the physiologically active red state^38,39^. A combination of spectroscopic and structural approaches revealed multiple features of these intermediates. Yet, the mechanism of OCP photoactivation is still far from being clear. Since in the active state the carotenoid evades contacts with Tyr-201 and Trp-288, many researchers have congruously proposed that the hydrogen bonds must break at some point and that this reaction initiates the following rearrangements of the OCP structure. In line with the static X-ray crystallography data of the basal OCP form and the individual carotenoid-bound NTD^40^, and a FRET-based study on the rhodamine-labeled full-length OCP^41^, it is assumed that the non-bonded (turned loose) carotenoid can slide from the CTD into the NTD, changing its position by more than 10 Å^36,39–41^, while the protein structure still stays compact. After that, protein–protein interactions become weakened, eventually leading to a complete separation of the CTD and NTD after the so-called N-terminal extension (NTE) detaches from its specific binding site on the CTD^42–44^ and unfolds as well as another short helical C-terminal tail (CTT)^45^. Detachment of NTE enables interactions of OCP with another regulator called the Fluorescence Recovery Protein (FRP), which properly reassembles the OCP domains to promote the reformation of the inactive orange state and, therefore, switching off the OCP-mediated phycobilisome quenching^46–50^. In the absence of FRP, photoactivation of OCP leads to an accumulation of long-lived active states, which can bind to the phycobilisome core and efficiently quench its fluorescence^51^. Thus, the interplay between OCP and FRP is an efficient way to control the primary photosynthetic activity.

As mentioned before, photoinduced conformational changes of OCP start with the disruption of hydrogen bonds between the keto-carotenoid and two specific residues (Tyr-201 and Trp-288, see Fig. 1A). It was proposed that this reaction is stimulated by a transition of the carotenoid into a specific state denoted S*, which is characterized by an extended lifetime (~ 15 ps to 25 ps) compared to the optically forbidden S_1_ (~ 3.2 ps) and allowed S_2_ (~ 100 fs) states^39,52^. Signatures of S* were found in multiple recent transient absorption experiments, however, the quantum yield of this state is estimated to be far below 5%^39,52^. Although the involvement of the enigmatic S* state in the primary stages of OCP photoactivation appears very appealing, additional experimental approaches are necessary to prove or disprove a correlation with the breaking of hydrogen bonds on the same timescale (tens of picoseconds).

In this work, we take benefit of our previous observations that the fluorescence of Trp-288 is statically quenched by H-bonding to the carotenoid in the dark-adapted state^38^ and employ this phenomenon to follow the early OCP photocycle using ultrafast time-resolved fluorescence spectroscopy. However, as wild-type OCP harbors five tryptophans, substantial protein engineering was required in order to make exclusive use of Trp-288 fluorescence as a signature for different stages of the OCP photocycle. Following the fluorescence readout of a fully photoactive OCP variant with only the critical Trp-288 left in place, we identified the rate of the hydrogen bond breakage which allowed us to associate the formation of the first intermediate of the OCP photocycle with a specific carotenoid excited state.

## Materials and methods

### Protein expression and purification

The OCP-3FH variant of OCP from *Synechocystis* sp. PCC 6803 carrying amino acid substitutions W41**F**, W101**F**, W110**F** and W277**H** was produced by site-directed mutagenesis using the QuikChange II mutagenesis kit (Agilent, Santa Clara, USA). Aiming at the conservation of the photoswitching ability, the substitutions of Trp-41, Trp-101 and Trp-110 for phenylalanine (“3F”) were subsequently introduced, and only the additional substitution of Trp-277 for histidine (“3FH”), but not phenylalanine, preserved photoactivity. Expression and purification of holoforms of the 6xHis-tagged OCP and the 3FH variant thereof were performed in echinenone– (ECN–) and canthaxanthin– (CAN–) producing *Escherichia coli* cells essentially as described before^53^. The His-tag was identical in the protein forms used. The recombinant proteins were purified by metal-affinity and size-exclusion chromatography to electrophoretic homogeneity and stored at 4 °C in the presence of 1 mM sodium azide, which was removed by diafiltration before experiments if necessary.

### Analytical size-exclusion spectrochromatography (SESC)

The samples containing individual OCP WT or OCP-3FH were loaded on a Superdex 200 Increase 5/150 column (GE Healthcare) equilibrated by a 20 mM Tris–HCl buffer, pH 7.6, containing 150 mM NaCl and operated at a 0.45 ml/min flow rate using a Varian 335/Varian 363 chromatography system (Varian Inc., Melbourne, Australia). During the run, full-spectrum absorbance was continuously recorded in the 240–850 nm range in 1 nm steps (4 nm slit width). The SESC profiles are shown only at specified wavelengths, whereas full absorbance spectra corresponding to the observed peaks are shown as extracted using the Galaxie Chromatography Data System (Agilent Technologies). Apparent Mw for the peaks were determined using column calibration with BSA dimer (132 kDa), BSA monomer (66 kDa), ovalbumin (43 kDa), and α-lactalbumin monomer (15 kDa). Expected Mw for a OCP WT monomer equals 34.6 kDa.

### Absorption measurements

Absorption spectra and time-courses were recorded by a spectrometer based on Maya 2000PRO (Ocean Optics, USA) CCD with 25-μm entrance slit using a stabilized deuterium UV–VIS light source (SL S204, Thorlabs, USA).

### Time-correlated single-photon counting (TCSPC) techniques for tryptophan fluorescence transients

In experiments on a timescale from milliseconds to seconds, OCP photoconversion was triggered by a blue LED (SOLIS-445C, Thorlabs, USA), the emission of which was additionally filtered by a bandpass filter (450 nm, 40 nm width, Thorlabs, USA) and focused onto the sample in a UV fused silica cuvette. The temperature of the sample was stabilized by a Peltier-controlled cuvette holder Qpod 2e (Quantum Northwest, USA) with a magnetic stirrer. Intrinsic tryptophan fluorescence of the protein was excited by a pulsed sub-nanosecond UV-LED (EPLED 265, Edinburgh Instruments, Scotland) with maximum emission at 270 nm corrected by a metal bandpass filter (270 nm, 15 nm width, Chroma, USA), delivering 700 ps pulses with average power of 0.6 μW at a 20 MHz repetition rate. Such low probe light power caused no changes in the level of Trp emission during long-term experiments. Fluorescence of the samples in the 300–400 nm range was collected perpendicular to the excitation beam path via a collimation lens coupled to the optical fiber of a 16-channel time-correlated single-photon counting spectrograph (PML-SPEC, Becker & Hickl GmbH, Germany). The spectrograph was equipped with a PML-16-C detector and a 1,200 lines/mm grating resulting in a resolution of 6.25 nm for each individual spectral channel. Fluorescence in 16 spectral channels was simultaneously recorded using a time- and wavelength-correlated single-photon counting (TCSPC) system based on a single TCSPC module (SPC-130EM, Becker & Hickl GmbH, Germany). The blue LED was driven by a LED driver (DC2200, Thorlabs, USA) which also provided TTL signals for synchronization with the TCSPC system. The TCSPC hardware was operated in the FIFO mode, thus recording a stream of spectrally tagged single photons along with external markers. In the measurement software (SPCM 9.82, https://www.becker-hickl.com/products/category/software/ Becker & Hickl GmbH, Germany), we used the so-called MCS_TA mode (multi-channel scalar triggered accumulation), which used the TTL signal from the LED driver as a reference trigger. This allowed for consecutive averaging (signal-to-noise increase) of multiple time-courses of Trp fluorescence intensity upon short (~ ms) actinic flashes.

In order to reach full contrast between the dark-adapted and photoactivated states, we studied the OCP photocycle at low temperatures (0 °C). Thus, taking advantage of slow conversion rates, we obtained a series of fluorescence decay kinetics upon photoactivation and subsequent back-conversion of the samples. Changes of fluorescence intensity, lifetime and spectrum were processed using the SPCImage 8.0 (https://www.becker-hickl.com/products/category/software/, Becker & Hickl, Germany) software package. To obtain the time-integrated fluorescence intensity and steady-state emission spectra, the number of photons in each time channel of individual fluorescence decay histograms was summed up after background noise correction (subtraction of dark offset measured in advance).

For femtosecond pump-probe measurements, tryptophan fluorescence was excited by femtosecond pulses at 262 nm obtained from the 4th harmonic (FHG) of a Yb femtosecond laser (TEMA-150 and AFsG-A, Avesta Project LTD., Moscow, Russia), driven at an 80 MHz repetition rate, delivering 150 fs pulses to the sample. The second harmonics (SHG) from the same optical generator (525 nm) was used as a pump pulse as its emission nicely overlaps with the absorption of the carotenoid in OCP. Time delays between the pump and probe pulses were controlled by an optical delay line (GCD-302002 M, DHC, China). Laser power was adjusted by neutral density filters and was typically 10 mW for the probe and 500 mW for the pump. The setup is visualized in Fig. 4A.

Due to the high power of actinic light and slow relaxation rates of photoactivated OCP, the measurements required circulation of the dark-adapted sample through an 860 μm diameter UV fused silica capillary. A peristaltic pump (PD 5101, Heidolph, Germany) provided a flow rate of 0.6 ml/s resulting in ~ 1 m/s displacement speed of the sample in the capillary. Optical pump and probe pulses were focused in the same spot (~ 100 µm) of the capillary. Tryptophan fluorescence was detected by a cooled ultrafast single-photon counting detector (HPM-100-07C, Becker & Hickl, Germany) with low dark count rate (~ 10 counts per second), coupled to a monochromator (ML-44, Solar, Belarus). The detection wavelength was set to 340 nm, which corresponds to the maximum of the tryptophan fluorescence emission spectrum in dark-adapted OCP. A difference between tryptophan fluorescence intensity with and without the pump beam was measured at each position of the optical delay line. Due to the low quantum yield of OCP photoconversion (~ 1.5%), measurement at each time point required 120 s in order to detect approximately 10^6^ photons, and this was repeated three times in order to achieve a reliable signal-to-noise ratio.

Measurements of steady-state fluorescence emission spectra used a CCD-based spectrometer (Maya2000 PRO, Ocean Optics, USA).

All calculations were performed using Origin Pro 9 (https://www.originlab.com/origin, OriginLab Corporation, USA). All experiments presented in this work were repeated at least three times.

## Results and discussion

### Elimination of all but one of the endogenous tryptophans leaves OCP photoactive

All Trp residues in OCP are highly conserved and (except W101) contribute to the so-called “carotenoid tunnel”, which provides a specific hydrophobic environment for the carotenoid (Fig. 1A). Thus, one could expect that mutations would significantly affect carotenoid binding efficiency, spectral properties and photoswitching. However, the combination of mutations W41F, W101F, W110F and W277H (resulting in a construct termed OCP-3FH) yielded a holo-protein with a visible absorption spectrum absolutely indistinguishable from the one of wild-type OCP (Fig. 1B). The absence of four out of five tryptophans entails a significantly decreased molar extinction of OCP-3FH in the UV region. Thus, the Vis/UV ratio increased from 1.85 (WT OCP) to 3.45 for the dark-adapted state of OCP-3FH (Fig. 1C, D and E). Since the absorption of WT OCP and the 3FH variant is identical in the visible region, we assumed that the molar extinction coefficient of the embedded carotenoid is equal in these two samples and estimated its contribution in the UV region to be equal to 12,340/M/cm in order to match the experimental values of Vis/UV absorption ratios of both samples. This means that the molar extinction of ECN in OCP is equal to 87,270/M/cm at 495 nm. Althougth the assumption about equal molar extinction of ECN in WT OCP and OCP-3FH might be oversimplified due to possible different carotenoid environment and/or local electric field effects, we would like to note that it is important to consider the contribution of the carotenoid to the absorption of OCP and OCP-like proteins in the UV region, since this critically affects the determination of OCP holoprotein concentration. Analytical SESC of OCP-3FH yields an Mw estimate of 34–35 kDa, which perfectly corresponds to the theoretical value (34.5 kDa) for a protein monomer (Fig. 1D and E). Since the hydrodynamic properties of OCP-3FH and wild-type OCP are identical, we conclude that the dark-adapted 3FH protein stays in a compact state, which is not disturbed by the introduced mutations.

Illumination of the OCP-3FH sample by actinic light causes a reversible transition to the red state, which can be monitored as an increase of absorbance at 550 nm (see Supplementary Fig. S1 for more details). Notably, back-conversion of OCP-3FH in the dark occurs approximately 50 times faster compared to WT OCP under the same experimental conditions (Supplementary Fig. S1). Very recently, we have demonstrated that OCP back-conversion can be drastically accelerated by covalent stabilization of protein–protein contacts, which prevents complete domain separation^54^, however, the mechanism in the OCP-3FH case is certainly different. The increased rate of back-conversion could likely be due to the mutations in the NTD and, especially, the W41F and W110F substitutions in the N-terminal part of the carotenoid binding tunnel. These substitutions presumably lower carotenoid-binding affinity in that region relative to the affinity of the CTD, which is almost unaffected by mutations. This implies that amino acid substitutions can be used also to control the directionality of carotenoid transfer.

The fact that the absorption of the sample reversibly decreases at 270 nm and increases at 320 nm upon photoconversion (see Fig. 1C) from the orange form (OCP^O^) to the red form (OCP^R^) proves that, in the 250–300 nm region, the absorption of amino acids overlaps with the UV absorption band of the embedded carotenoid, which should also be taken into account for the calculation of protein concentration.

At low temperatures, due to reduction of the back-conversion rate (Supplementary Fig. S1B), OCP-3FH can be converted into the red state completely, with an absorption spectrum similar to the one of photoactivated WT OCP. Similar to WT OCP, the absorption of OCP-3FH in the dark-adapted state represents a superposition of orange (77%) and red (23%) states (Fig. 1B) according to spectral decomposition^55^. Importantly, according to the SESC data, the red-absorbing state of the dark-adapted sample, which, as we assume, is spectrally indistinguishable from OCP^R^, should be attributed to a compact 35-kDa protein state with structural features of the dark-adapted OCP^O^, in which spontaneous conformational changes of the embedded carotenoid may occur.

Combining these observations with previous data on the OCP-W288A variant, which can form a compact orange state as well^38^, we infer that none of the endogenous tryptophans of OCP is absolutely essential for the photoactivity of OCP, even though these residues might be important for the regulation of photoprotection via prolonging the lifetime of the red active state^56^. Thus, OCP-3FH represents a valid model for the investigation of OCP photocycling phenomena with only a single, functionally most critical, Trp residue preserved. The drastically increased rate of back-conversion of photoconverted OCP-3FH suggests that the dynamic aspects of functional interactions with the phycobilisome or FRP are clearly different; however, it is out of the scope of the current study, which focuses on OCP photocycling.

### Single Trp fluorescence in OCP-3FH is sensitive to H-bonding between Trp-288 and carotenoid

**Figure 2:**
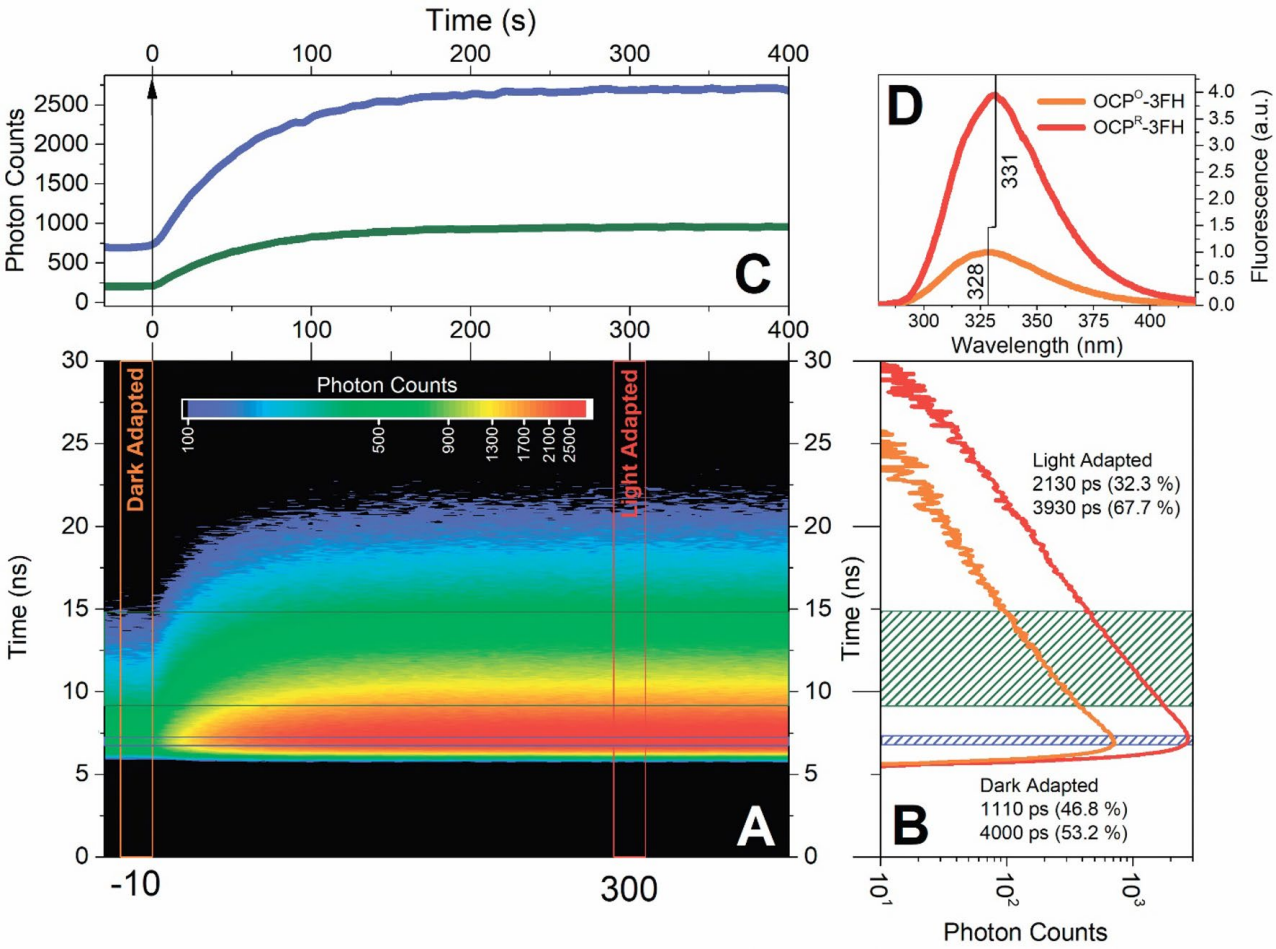
Accumulation of the red state of OCP-3FH as revealed by time-resolved Trp fluorescence spectroscopy. The color-coded image (**A**) represents a sequence of fluorescence decay kinetics of the single Trp in OCP-3FH recorded with an integration time of 5 s. Zero on the horizontal timescale represents the beginning of photoactivation by a 5 mW LED emitting at 445 nm. The vertical orange and red cross-sections indicate intervals (five neighboring decay curves) in which photons were selected to show the characteristic Trp-288 fluorescence decay of OCP-3FH in the dark and light-adapted states. (**B**) Shows these decays on a semilogarithmic scale (logarithm of photon number vs. linear timescale) and corresponds to the color-coded image. The horizontal cross-sections show how the number of photons changes upon photoactivation of the sample at different parts of fluorescence decay and the corresponding time-gated intensity time-courses (**C**): the blue line shows changes at the peak intensity [blue area in panel (**B**), average of 10 time-channels, total width ~ 488 ps] of the decay, while the green line shows changes in time channels which correspond to the slow component of the decay [green area in panel (**B**), average of 100 time-channels, total width ~ 4.8 ns]. Experiments were conducted at 0 °C. Sample concentration was equal to 2 µM. Each point represents an average of four independent experiments. (**D**) Steady-state fluorescence spectra of OCP-3FH in the dark- (orange line) and light-adapted (red line) states. See Supplementary Fig. S4 for additional data analysis and a comparison of Trp-288 fluorescence and ECN absorption changes.

We found that Trp fluorescence emission is low in the dark-adapted state of the OCP-3FH holoprotein, and that photoactivation at low temperature leads to a fourfold increase of fluorescence intensity due to the accumulation of the red state (Fig. 2). This increase in fluorescence intensity is accompanied by a 3 nm red-shift of the maximum of the Trp fluorescence spectrum (Fig. 2D) and an increase in the amplitude-weighted average fluorescence lifetime of the single Trp-288 (from 2.65 to 3.35 ns, components of the decay are shown in Fig. 2B). In our previous work on WT OCP, we assigned dynamic and static quenching of Trp fluorescence in the dark-adapted state to different Trp residues and determined that Trp-288 fluorescence in OCP^O^ is statically quenched^38^, which is in excellent agreement with the present observations. Since a 21% increase in the average lifetime cannot account for the fourfold (300%) increase of fluorescence intensity, we assume that it is the number of non-quenched Trp-288 residues that increases upon photoactivation. In other words, photoconversion of OCP eliminates static quenching of Trp-288 fluorescence. This raises the question why the Trp-288 fluorescence is quenched in the dark-adapted state substantially, but not completely.

It is generally accepted that Trp fluorescence exhibits multiexponential decay and is highly sensitive to the microenvironment^57^. Two characteristic lifetimes are usually detected for Trp in polar environment^58^. Conventionally, changes in Trp fluorescence intensity and lifetime are associated with quenching of the excited *La* state^59^, the energy of which is known to be sensitive to hydrogen bonds. The formation of a hydrogen bond by Trp displays a red shift in fluorescence spectra^60^. Quenching of the *La* state can be achieved by electron transfer from the excited Trp to an amide carbonyl group^61,62^, or by various quenchers^57,63^. In addition, some investigations attributed the Trp fluorescence parameters with its rotameric states in the protein fold^64^; however, this concept is still questionable^58,65,66^. We assume that Trp-288 fluorescence quenching in OCP-3FH is mainly caused by two separate energy distribution pathways: formation of the charge transfer state (CT) and excitation energy transfer (EET) to the S_2_ state of the carotenoid (Supplementary Fig. S3). The CT state is an excited state in which electron density migrates from the indole ring to the amide carbonyl group. Such charge re-distribution gives rise to a large dipole moment of the CT state; thus, the CT state’s energy is highly dependent on the local electric field. The strong (~ 2.1 Å) hydrogen bond between Trp-288 and the keto-oxygen of the carotenoid obviously orients the negative charge towards the indole ring. This effect can additionally be aggravated by Tyr-201 in the local environment (see Fig. 1A). In this situation, the CT state will be energetically stabilized and, therefore, become favored over the *La* state, which, in effect, will cause rapid transition into the non-radiative CT state. Breakage of the hydrogen bond is expected to cause the opposite effect: destabilization of the CT relative to the *La* state. The resultant reduction of the efficiency the CT formation will lead to an increase of the Trp fluorescence signal.

Furthermore, sample heterogeneity must be considered as a possible reason for Trp emission in the dark-adapted state. It is important to note that the OCP-3FH protein preparation is free from apoprotein (Fig. 1), which, of course, would otherwise contribute to Trp fluorescence emission. Indeed, the increased hydrodynamic size of the apo-form allows for complete chromatographic separation from the compact holo-form of the protein^67^, which excludes that the apo-form contributes to the observed Trp emission. Next, as proposed earlier^38^, the static quenching of Trp-288 in the orange state could be due to interactions with the keto-oxygen of ECN (e.g. by the formation of a hydrogen bond), as inferred from crystal structures^34^. When the hydrogen bond is broken, this type of quenching should be eliminated upon photoactivation and conversion of the sample into the final red state, in which the carotenoid is translocated into the NTD and separated from Trp-288 due to domain separation. Considering that all OCP-3FH molecules are converted into the OCP^R^ state and that the fluorescence lifetime of Trp-288 changes from 2.65 to 3.35 ns, we can estimate the fraction of Trp-288 residues emitting in the compact dark-adapted state as 28.1%. This number fairly corresponds to the spectral heterogeneity of the carotenoprotein in the dark-adapted state, since a spectrally red-absorbing form is observed in the UV–Vis absorption spectrum with a contribution of 23% (Fig. 1B). We suggest that this red-absorbing species is characterized by the absence of a hydrogen bond between Trp-288/Tyr-201 and the keto-oxygen of ECN. Thus, spontaneous (and reversible) disruption of the hydrogen bond, possibly entailing isomerization of the carotenoid and transition into a red-absorbing state, might already occur in the compact protein state (see Fig. 1D and E) with an OCP^O^-like structure. Given that OCP can be orange and photoactive even in the absence of Trp-288^38^, we assume that in a *compact red* state, the hydrogen bond between the keto-carotenoid and Tyr-201 is also broken, while in the orange state it should be intact. This also suggests that in ~ 5% of the sample, ECN is lacking hydrogen bond with Trp-288, while it is kept in the orange state due to hydrogen bond with Tyr-201.

Postulating that ~ 71.9% of dark-adapted OCP-3FH species are orange and “invisible” in terms of Trp fluorescence, we conclude that it is possible to follow the disruption of ECN-Trp hydrogen bond by monitoring the appearance of Trp-288 emission in OCP-3FH.

### Intermediates of OCP photocycle revealed by time-resolved Trp-288 fluorimetry

**Figure 3:**
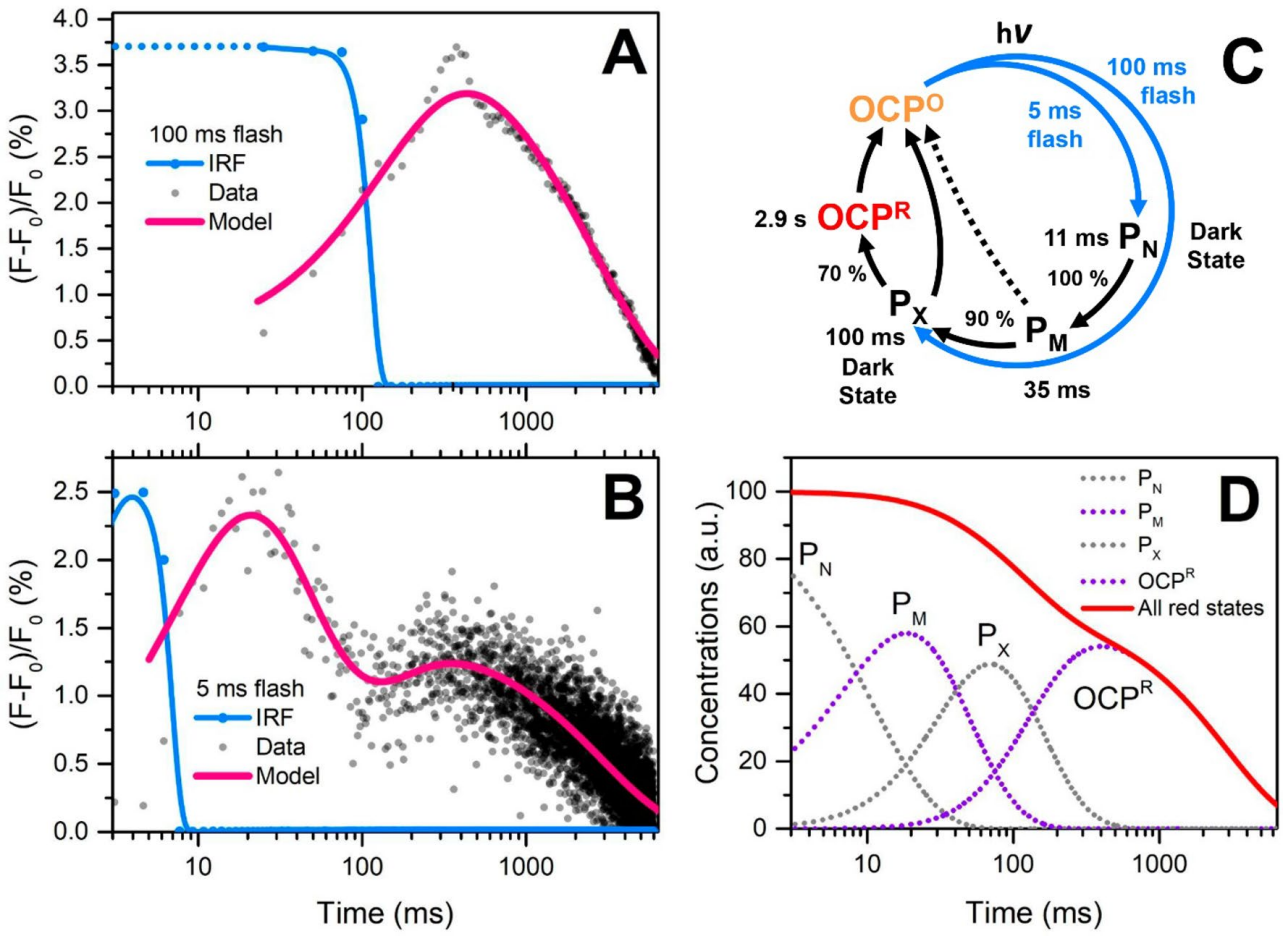
Characteristic time courses of Trp-288 fluorescence intensity in OCP-3FH induced by 100 ms (**A**) and 5 ms (**B**) actinic flashes (blue LED) measured at 20 °C. Trp fluorescence was excited by 262 nm and measured at 340 ± 10 nm in a time-correlated single-photon counting regime. The fluorescence intensity measured before the flash (${F}_{0}$) was subtracted from each point in order to estimate the relative increase of fluorescence intensity as $(F-{F}_{0})/{F}_{0}$. Each time course represents averaging of ~ 2000 photocycles. At least 2 × 10^5^ photons were collected for each time step. The signal was recordered with time steps of 25 ms in (**A**) and 1.5 ms in (**B**). Sample concentration was equal to 2 µM. (**C**) A model of the OCP photocycle derived from Trp fluorescence experiments. Numbers indicate kinetic parameters that were used in order to fit experimental data in panels (**A**) and (**B**) (pink lines) by a sequential model. (**D**) Relative concentrations of intermediate states generated after 5 ms flash (experiment in panel **B**).

The conventional way to characterize the OCP photocycle is to illuminate the sample with powerful actinic light for tens of seconds or even minutes while recording the changes of absorption at 550 nm. This approach allows for the accumulation of high concentrations of the long-living final red state OCP^R^ with separated protein domains, and thus it is suitable for investigation of physiologically relevant states. On the other hand, continuous illumination makes it impossible to study, in particular, the early intermediates of the OCP photocycle which appear already on a millisecond or even shorter timescales. A drawback of shortening the actinic flash is obviously the loss of contrast between the states. This problem is especially pronounced due to the low quantum yield of the final red OCP state, which is below 0.5%^39^. In order to eliminate this problem, we used periodic excitation of OCP-3FH by relatively short actinic flashes with consequent averaging of Trp fluorescence intensity time-courses (Fig. 3). This approach allowed us to see a multistage switching of the Trp-288 state in the course of the OCP photocycle.

In Fig. 3, we present the relative increase of Trp-288 fluorescence intensity on a logarithmic timescale, in order to show that Trp fluorescence intensity reaches its maximum value long after the actinic flash ended. In order to explain such a complex kinetic behavior, we have to consider that after exposure to actinic light on a ms timescale, the Trp-288 residue in the protein passes through several intermediate states (sequence termed P_N_-P_M_-P_X_-OCP^R^ herein, see Fig. 3C), the fluorescence intensity of which is proportional to the concentration multiplied by fluorescence quantum yield, which might be different for each state. Explicitly, we have to assume that the fluorescence quantum yield of the P_N_ intermediate is equal to 0, while such of P_X_ state is 4 times lower compared to OCP^R^. Without these assumptions it is impossible to describe the increasing intensity of Trp fluorescence 30 ms and 300 ms after the actinic flash was turned off. At the moment it is impossible to determine Trp fluorescence lifetimes or relative changes of the Trp emission maximum to answer questions about the actual reason of fluorescence intensity changes upon transition from one intermediate to another, so we cannot exclude both possibilities, which does not refute the existence of several distinct intermediates on the timescale of 100 ms.

The nature and number of the discovered intermediates (P_N_–P_M_–P_X_) detected by Trp-288 fluorescence during the transition from OCP^O^ to OCP^R^ is puzzling. It is probable that only the final state with 2.9 s lifetime represents OCP^R^ with separated domains, while the other states, which evolve and disappear significantly faster (Fig. 3), probably represent OCP states in which the structural domains are still positioned like in the compact, dark-adapted state, thus, notably, providing an intact carotenoid tunnel and ensuring that the carotenoid could rapidly slide back into the CTD. Thus, conformational motions on the 100 ms timescale can be assigned to slow final steps of OCP photoconversion, which are related to changes in protein conformation. Recently, Konold et al. combined time-resolved transient absorption and IR spectroscopy to study the OCP photocycle in the time range from ~ 100 fs to 750 µs. These authors described four intermediates including an intermediate P3 appearing within 10 µs after the actinic flash with an absorption very similar to the final OCP^R^ state, which was not further evolving until the last time delay (750 µs). It was proposed that P3 represents a state with the carotenoid already translocated into the NTD, while the NTE and the C-terminal tail (CTT) helices were intact and have to unfold and/or dissociate from the C-terminal β-sheet on time scales longer than 0.5 ms in order to allow domain separation to take place^39^. In this regard, we suggest that P3 corresponds to the P_N_ state of Trp-288 (see Fig. 3), while the two following stages (P_M_–P_X_) are associated with conformational changes of the NTE and CTT. Although the question about the reduced Trp fluorescence intensity of specific intermediate states (P_N_ and P_X_) remains unanswered, we would like to note here that Trp-288 is located in a β-sheet of the CTD, which forms contacts with both the NTE and CTT (see Fig. 1A). Considering that the conformation of this region is significantly affected by photoactivation and has been suggested to be responsible for OCP^R^ dimerization at high protein concentration, we assume that Trp-288 might be sensitive to such changes of the local environment. It is also important to note that hydrogen bonds with Trp-288 are disrupted on a significantly shorter time scale, so it is impossible to relate the observed slow changes of Trp-288 fluorescence to interactions with ECN (like induction of CT states); however, a decrease of the efficiency of EET from Trp to ECN, in the course of an increasing distance as the domains separate, might contribute to the observed effects (Fig. 3A,B and Supplementary Fig. S3).

**Figure 4:**
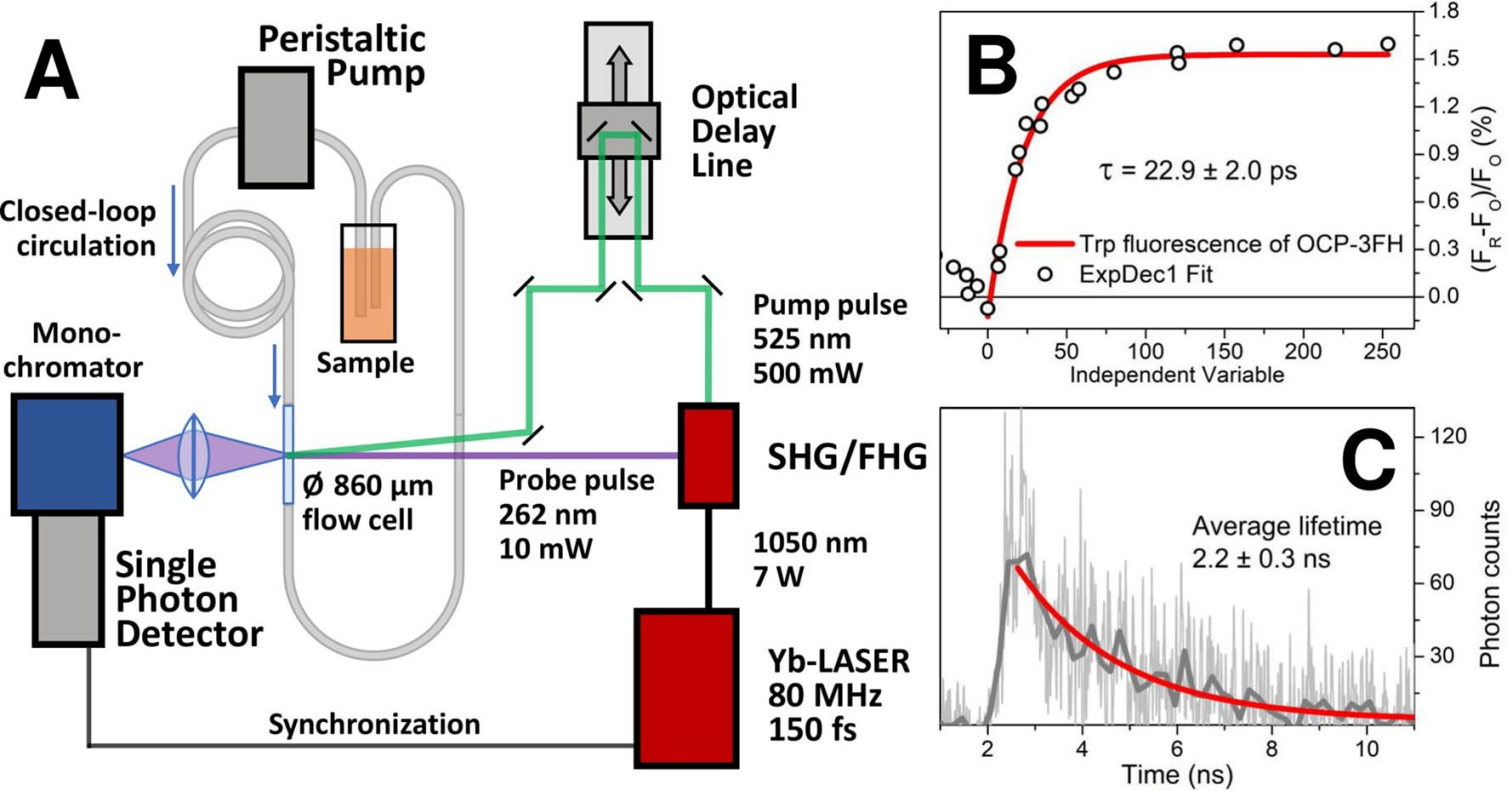
(**A**) An experimental setup for femtosecond pump-probe fluorescence spectroscopy, see “Materials and methods” section for detailed explanation. (**B**) Transient increase of the Trp-288 fluorescence intensity caused by photoexcitation of OCP-3FH by a single 150-fs actinic flash. Each point is an average of three measurements. (**C**) Fluorescence decay of Trp species appearing after excitation of OCP-3FH with the pump pulse averaged for delay times between 100 and 250 ps.

**Figure 5:**
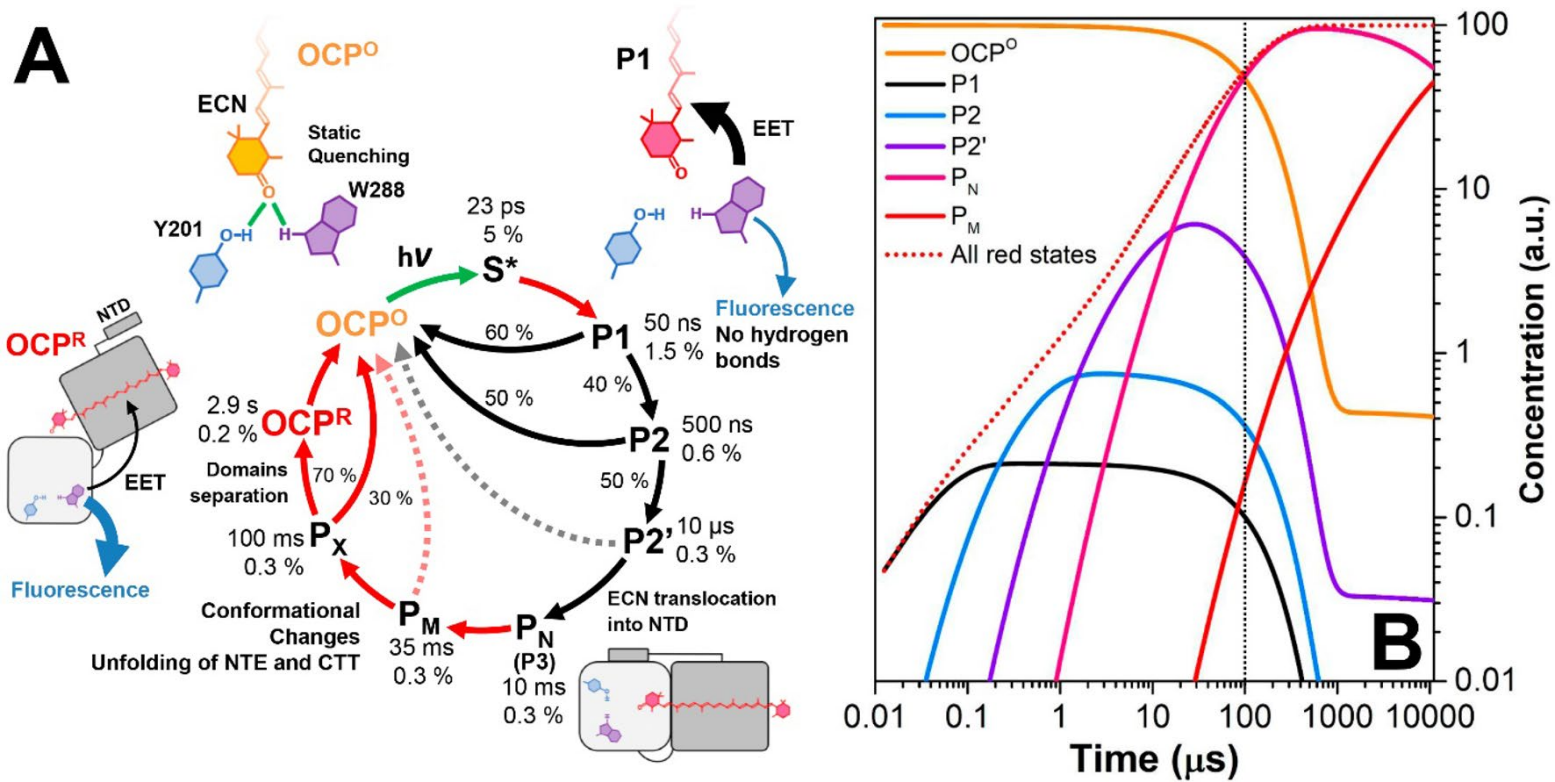
(**A**) A working model of photoinduced transitions in OCP showing the main intermediates of the photocycle. The model is based on the kinetic characteristics of the S* state of ECN and the intermediates (P1–P3) of the OCP photocycle revealed by time-resolved transient absorption and IR spectroscopy by Konold et al.^39^ , and intermediates P_N_-P_X_ revealed by Trp-288 fluorescence in the current work (see Fig. 3). Red arrows indicate processes studied in this work (see Figs. 3 and 5). The essential structural characteristics of the most important states are presented schematically. In the structures, hydrogen bonds are shown in green, excitation energy transfer (EET) from Trp-288 to ECN is shown in black and Trp-288 fluorescence is shown in blue. The probability of EET and fluorescence is represented by the thickness of black and blue arrows. (**B**) Estimation of relative concentrations of intermediate states P1-P_M_ generated upon quasi-continuous illumination of 2 µM OCP by 500 mW (average power) laser flashes (80 MHz, 150 fs, 525 nm) calculated in the framework of the model presented in panel (**A**). Red dotted line represents total concentration of red photoproducts.

At this point, we questioned how rapidly the hydrogen bond between the ECN and Trp-288 is broken upon photoexcitation of the carotenoid. To answer this question, we constructed a pump-probe setup (Fig. 4A) with rapidly flowing (~ 1 m/s) sample. It should be noted that due to the high repetition rate of our laser system (80 MHz) the sample could be pumped and probed multiple times during ~ 100 μs which pass to completely replenish the sample in the laser focus/probe spot. This means that the detected Trp-288 fluorescence might originate from a mixture of photoproducts. In order to estimate the contributions of intermediates in such an experiment, we elaborated a kinetic model (Fig. 5A) based on the properties of OCP photocycle intermediates described in the literature^38,39^. Considering the kinetic characteristics of intermediates P1-P_M_, in particular the experimental parameters of excitation (flux) and concentration of OCP^O^ (2 µM), we estimated the concentration of P1 in our experiment to be about 3 nM, while the concentration of P2, P2′, P_N_ (or P3) and P_M_ is 10 nM, 97 nM, 490 nM and 1 nM, respectively (see Fig. 5B). Thus, assuming that only P1 concentration might change on the 100 ps time scale, we expected to detect the time-course of this intermediate on top of a huge increase of the background signal, which results from the red intermediate states with lifetimes of 500 ns and longer and accumulate upon repetitive actinic flashes in addition to the red states which already existed before each flash. Surprisingly, we detected no significant increase in the background level of the Trp-288 fluorescence, although upon increasing the delay between the pump and probe pulses, we observed a gradual increase of fluorescence intensity, which reached a plateau with an extra ~ 1.5% of the initial signal (Fig. 4B). Once again, we note that the intensity of Trp-288 fluorescence is proportional to the concentration of specific state(s) and their fluorescence quantum yield. In this regard, as we assume that the concentrations of P2-P_N_ states are significant compared to P1, we must consider that Trp-288 in states P2 and P2′ is also quenched, similar to the P_N_ (or P3) state (see Fig. 3 and description). However, we cannot exclude that the yields of these intermediates in WT OCP and OCP-3FH mutant are completely different. Indeed, if the introduced mutations make the OCP^R^ → OCP^O^ relaxation at least tenfold faster (see Supplementary Fig. S1), the rates of the P2 → OCP^O^ and P2′ → OCP^O^ reactions might be increased as well.

The rise of the Trp-288 fluorescence intensity, which we assign to accumulation of the P1 state, occurs with a time constant equal to 22.9 ± 2.0 ps. Compared to retinal photoisomerization in bacteriorhodopsin, which occurs on a sub-picosecond timescale^68^, the formation of the photoproduct in OCP is very slow, which suggests a completely different reaction mechanism. Konold et al. proposed that the population of the so-called S* carotenoid state, which the authors interpreted as a structurally distorted form of the carotenoid’s S_1_ state, strains the hydrogen bonds to Trp-288 and Tyr-201, and eventually results in their breakage with a low yield of ~ 1.5%^39^. The lifetime of the S* state was estimated by Konold et al. and Kuznetsova et al. to be in the range from 13 to 25 ps^39,52^, which is in good agreement with our observations supporting that the population of the S* state might precede both, the breakage of the hydrogen bonds and formation of the first intermediate of OCP photocycle, and may be critical for its evolution.

Intermediates appearing in our experiment after excitation of the OCP-3FH sample with a 150-fs flash tend to have slightly shorter average Trp-288 fluorescence lifetimes (Fig. 4C) compared to the dark-adapted sample (see Fig. 2B); however, the low number of photons in differential traces (Fig. 4C) does not permit analysis of individual components. We suggest that changes in the Trp fluorescence lifetimes might at least partially be associated with different excitation energy transfer (EET) efficiencies. Considering FRET as a mechanism of Trp fluorescence quenching together with the available structural data, it is reasonable to assume that the lowest EET efficiency is expected for the final OCP^R^ state with the separated domains, in which Trp and ECN are at a significant distance with randomly oriented transition dipoles. In this regard, the higher EET efficiency in the intermediate which appears on a picosecond timescale is reasonable since the protein should remain in a compact state. Considering FRET as a possible mechanism of EET and estimating the orientation factor (*k*^2^) based on the the relative orientation of the transition dipoles in the available structure (PDB: 3MG1) results in *k*^2^ values equal to 1.47, which together with a short donor–acceptor distance entails an EET efficiency of ~ 97.5% and results in < 50 ps components in Trp fluorescence decay. Although it is currently impossible to resolve the fast Trp fluorescence decay components during the first 100 ps after an actinic flash, the relatively high Trp fluorescence lifetimes observed in OCP-3FH indicate that the spontaneous disruption of hydrogen bonds is followed by relaxation of protein conformation, which probably affects both, mutual orientation of Trp and ECN transition dipole moments and the distance. Given that large-scale rearrangements like the 12 Å shift of ECN into the NTD^40^ occur within ~ 10 µs after photoexcitation^39^ and must lead to a significant decrease of EET efficiency (see Supplementary Figs. S2, S3), which is in contrast to the need to consider a “dark” Trp-288 state for explaining the experimental data in Figs. 3 and 4, we postulate that Trp fluorescence might be sensitive not only to the presence (or absence) of the hydrogen bonds with ECN and the donor–acceptor distance, but also to sequential conformational rearrengements of the protein matrix. Thus, these changes of Trp fluorescence quantum yield should be assigned to specific structural determinants.

## Conclusions

In this work, we constructed a photoactive variant of the orange carotenoid protein with only a single Trp residue (Trp-288) to use fluorescence emission of this residue as a reporter for the state of the protein, its carotenoid cofactor, and explicitly the hydrogen bond with the keto-carotenoid. Spectroscopic techniques with time resolution of up to 100 fs were applied to study the photocycle of this OCP-3FH variant. Though the introduced mutations caused an increase of the OCP^R^ state relaxation rate, they had no effect on the ability to photoswitch or carotenoid absorption, thus making OCP-3FH an interesting model for spectroscopic approaches to dissect the photocycle. Analysis of the intrinsic fluorescence of the single critical Trp-288 as a probe revealed several aspects of OCP functioning. The spectral heterogeneity of carotenoid absorption, which is observed even in the compact dark-adapted protein state arises from spontaneous disruption of hydrogen bonds between ECN and Trp-288/Tyr-201. This implies that approximately 25% of “dark-adapted” OCP is in some compact “red” state, in which the carotenoid gains conformational freedom due to the loss of H-bonding to Trp-288/Tyr-201. However, the probability that these “dark-adapted” “red” states evolve into the physiologically active OCP^R^ state with separated protein domains is low, since hydrogen bonds eventually reorganize fast. Using pump-probe techniques we found that breaking of the ECN-Trp-288 hydrogen bond occurs with a time constant of 22.9 ± 2.0 ps, in line with the recent suggestion that this reaction is initiated by a transition of the ECN cofactor into the so-called S* state with a time constant of 14–25 ps^39,52^, which is significantly longer than the one of the S_1_ state (~ 3.2 ps). Altogether, these findings prove that the first intermediate of the OCP photocycle, for which the structure of carotenoid is substantially different from the dark-adapted state, appears on a picosecond timescale. By kinetic analysis of Trp fluorescence, additional intermediate states were revealed on the 100 ms time scale, and estimates for the rate of protein domains separation and formation of the physiologically active state OCP^R^ are provided.We assume that further experimental refinement for precise measurements of Trp-288 fluorescence lifetimes will be rewarding in particular for tracking carotenoid translocation between the OCP domains, and in order to clarify the nature of the very early OCP photocycle intermediates in the future.

## Supplementary information

Supplementary figures.
