# Supplementary material for: Probing of carotenoid-tryptophan hydrogen bonding dynamics in the single-tryptophan photoactive Orange Carotenoid Protein
Source: Sci Rep. 2020 Jul 16;10:11729. doi: 10.1038/s41598-020-68463-8 (PMC7366913; doi:10.1038/s41598-020-68463-8)
Supplement: Supplementary file 1 — Supplementary figures. [file 41598_2020_68463_MOESM1_ESM.docx]

**Supplementary Information**

**Probing of carotenoid-tryptophan hydrogen bonding dynamics in the single-tryptophan photoactive Orange Carotenoid Protein**

*Eugene G. Maksimov^1,2^, Elena A. Protasova^1^, Georgy V. Tsoraev^1^, Igor A. Yaroshevich^1^, Anton I. Maydykovskiy^3^, Evgeny A. Shirshin^3^, Timofey S. Gostev^1^, Alexander Jelzow^5^, Marcus Moldenhauer^4^, Yury B. Slonimskiy^2^, Nikolai N. Sluchanko^1,2^, Thomas Friedrich^4^

*^1^Lomonosov Moscow State University, Department of Biophysics, Faculty of Biology, 119991, Moscow, Russia.*

*^2^A.N. Bach Institute of Biochemistry, Federal Research Center of Biotechnology of the Russian Academy of Sciences, 119071, Moscow, Russia.*

*^3^Department of Quantum Electronics, Faculty of Physics, M.V. Lomonosov Moscow State University, 119992, Moscow, Russia*

*^4^Technical University of Berlin, Institute of Chemistry PC 14, Straße des des 17. Juni 135, D-10623, Berlin, Germany.*

*^5^Becker & Hickl GmbH, Nunsdorfer Ring 7-9, 12277 Berlin, Germany*

*Correspondence and requests for materials should be addressed to E.G.M.

(e-mail: emaksimoff@yandex.ru)


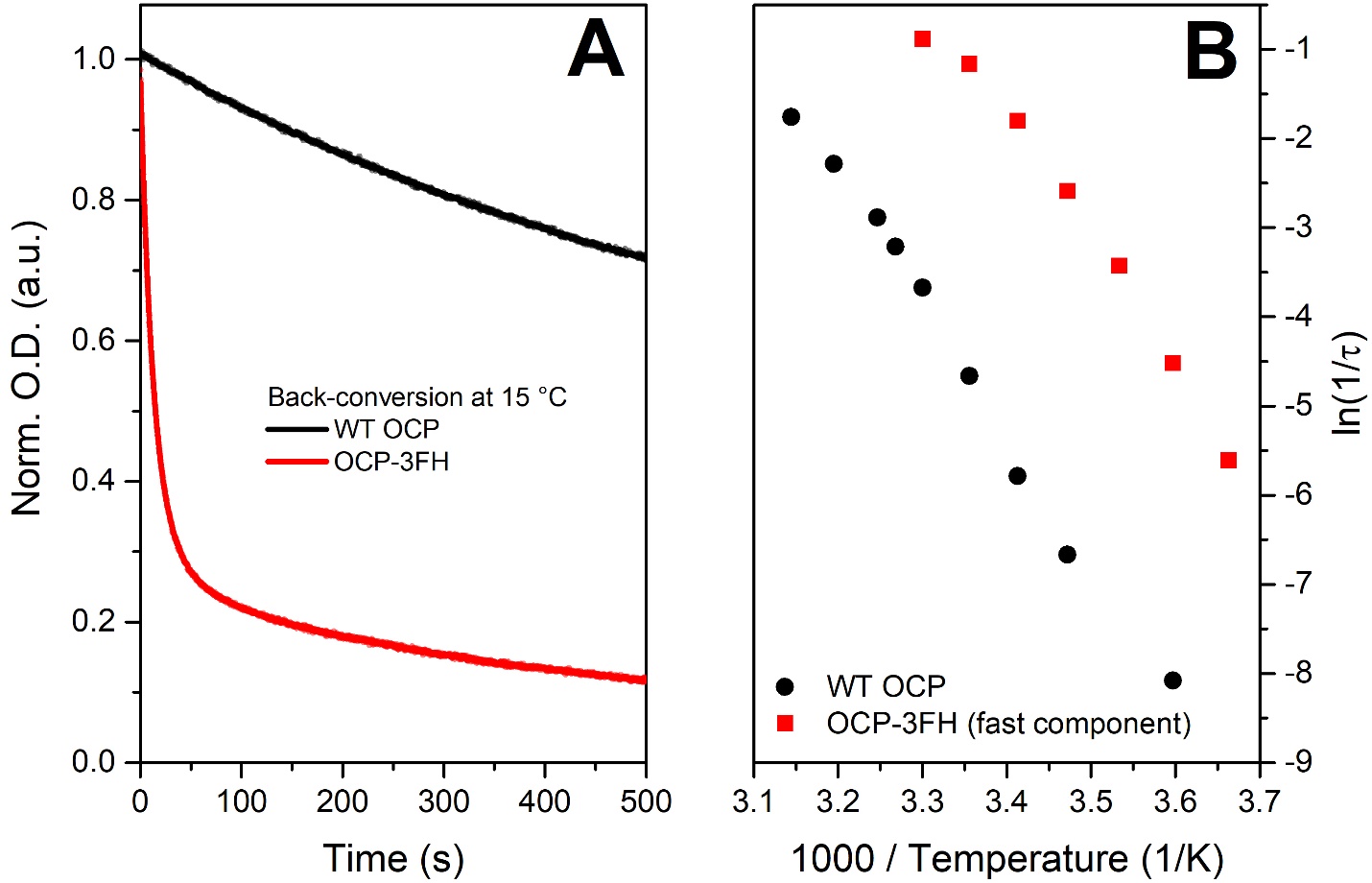


**Figure S1**. (**A**) – characteristic time-courses of WT OCP (black) and OCP-3FH (red) relaxation measured as changes in optical density at 550 nm and 15 °C after exposure to actinic light (blue LED, 200 mW, 120 s). Protein concentration was 10 µM. (**B**) – Arrhenius plots for the OCP^R^-OCP^O^ relaxation rates of WT OCP (black) and OCP-3FH (red). For OCP-3FH, the fast component was considered as OCP^R^‑OCP^O^ relaxation.

While checking the photoactivity of the OCP-3FH on the 1-100 s timescale, we noticed that unlike the wild-type protein, the relaxation of OCP-3FH was not monoexponential. Although a major fraction of photoconverted OCP-3FH relaxed approximately 50 times faster than WT OCP, relaxation of the minor part was slow (*Figure S1A*). We found that the yield of these long-living states was increasing during long exposures of the sample to actinic light and resulted in red-shifted difference (light-adapted minus dark-adapted) absorption spectra (*Figure S2A*), which does not occur for WT OCP. Similar red-shifted difference absorption spectra were observed in our experiments, in which the absorption of the sample was measured upon delivery of the carotenoid from dimeric CTDs of OCP (so-called COCP) to apo-OCP, resulting in the appearance of the regular, orange form OCP (Maksimov, E. G., et al. (2017). "The unique protein-to-protein carotenoid transfer mechanism." Biophysical journal: DOI: 10.1016/j.bpj.2017.1006.1002.) and upon delivery of the carotenoid from photoactivated OCP into the homologue of CTD from *Anabaena* (Slonimskiy, Y. B., et al. (2019). "Light-controlled carotenoid transfer between water-soluble proteins related to cyanobacterial photoprotection." Febs j 286(10): 1908-1924. DOI: 10.1111/febs.14803). Absorption of both, COCP and CTDH, is red-shifted compared to OCP^R^ by 10-30 nm (depending on carotenoid type), making the sample color purple or even violet. According to our previous data, full-length OCPs can also form 69 kDa dimers with violet color, if for some reasons one carotenoid molecule is shared by two CTDs (for more details, see Maksimov et al. (2017). Biophysical journal: DOI: 10.1016/j.bpj.2017.1006.1002.). We assume that spectroscopic signatures of “violet” forms observed in experiments with OCP-3FH could be due to the mutations in the NTD, which are expected to reduce the affinity of carotenoid binding in that region, while the CTD remains unchanged, which results in relatively increased stability of CTD-CTD interactions. Since the focus of this work was to study the photoactivation process of OCP in temporal detail, our goal was to identify conditions, under which the yield of such violet forms is largely suppressed. We found that the accumulation of violet forms could be reduced by decreasing the temperature (*Figure S2B* and *C*). Due to this fact and since it is favorable for the intended kinetic analyses to slow down photoconversion in order to facilitate the employment of increased integration times (for time-resolved fluorescence measurements), some experiments were carried out at 0 °C, subsequently (ensuring that the sample remains in the non-frozen state due to the presence of 150 mM NaCl).


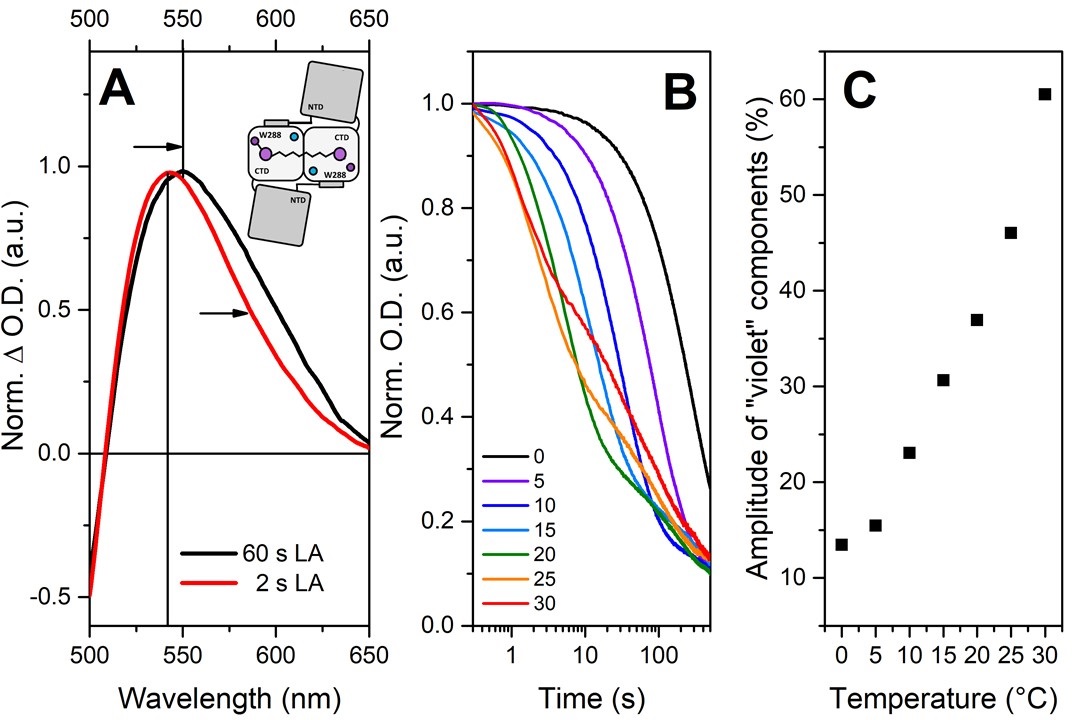


**Figure S2.** Photoinduced accumulation of violet forms in OCP-3FH samples and consequent relaxation. (**A**) – difference (light-adapted (LA) minus dark-adapted) absorption spectra of OCP-3FH after 2 s (red) and 60 s (black) exposure to actinic light (blue LED, 200 mW). Arrows indicate that the absorption of the photoproduct appearing upon prolonged exposures is red-shifted. The experiment was conducted at 25 °C. Inset shows a schematic representation of a possible OCP-3FH dimer based on CTD-CTD interactions. (**B**) - time-courses of OCP-3FH relaxation measured as changes in optical density at 550 nm at different temperatures (indicated by numbers in °C) after exposure to actinic light (blue LED, 200 mW, 120 s). Protein concentration was 10 µM. Time-courses were approximated by a sum of exponentially decaying functions, which allowed estimation of relative yield of the fast decaying “red” and the long-living “violet” fraction. (**C**) – dependency of the relative yield of long-living “violet” fraction on temperature.


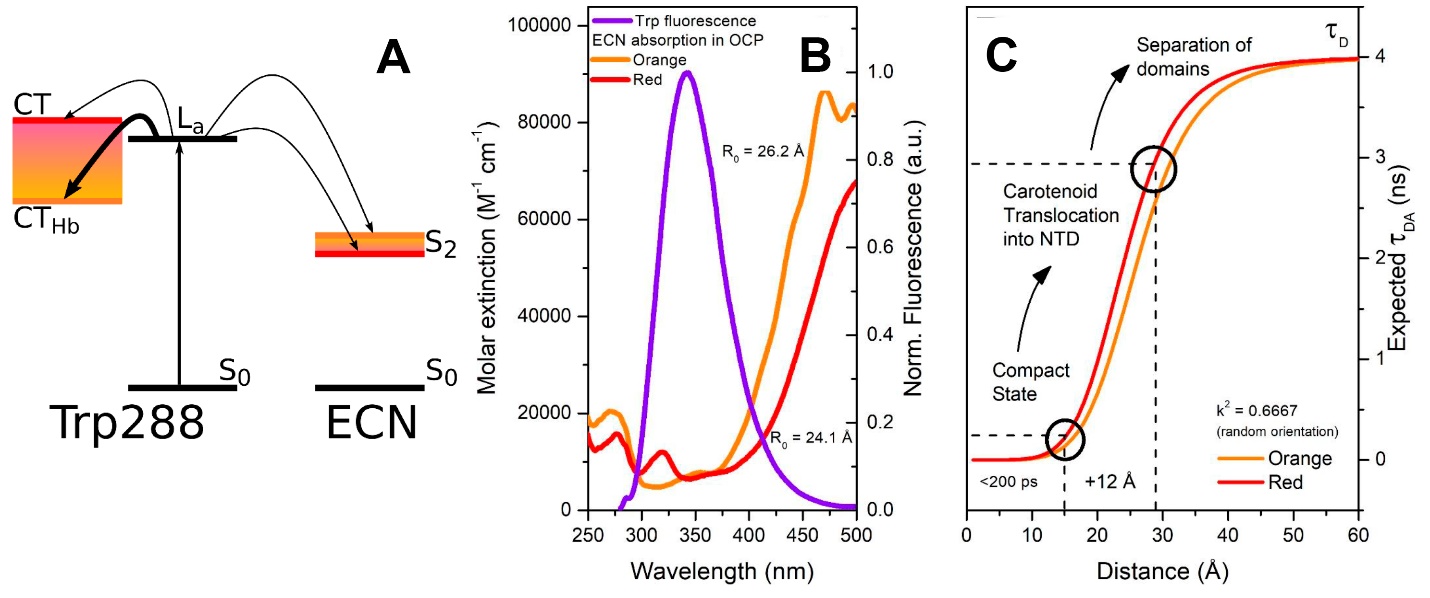


**Figure S3.** (**A**) – schematic representation of energetic interplay of the *L_a_* state with the Charge Transfer state (CT) and the S_2_ state of the carotenoid (ECN). The position of the corresponding energy levels upon photoconversion of OCP from the dark-adapted orange state (with hydrogen bonds, Hb) into the red state is indicated by color. (**B**) – overlap of Trp emission and absorption of ECN in the orange and red state of OCP. (**C**) – expected donor lifetime as a function of the center-of-mass distance between energy donor and acceptor in frames of Förster resonance energy transfer formalism. Lifetimes were calculated considering the corresponding $R_{0}$ values and assuming $\tau_{D}$ to be equal to 4 ns.


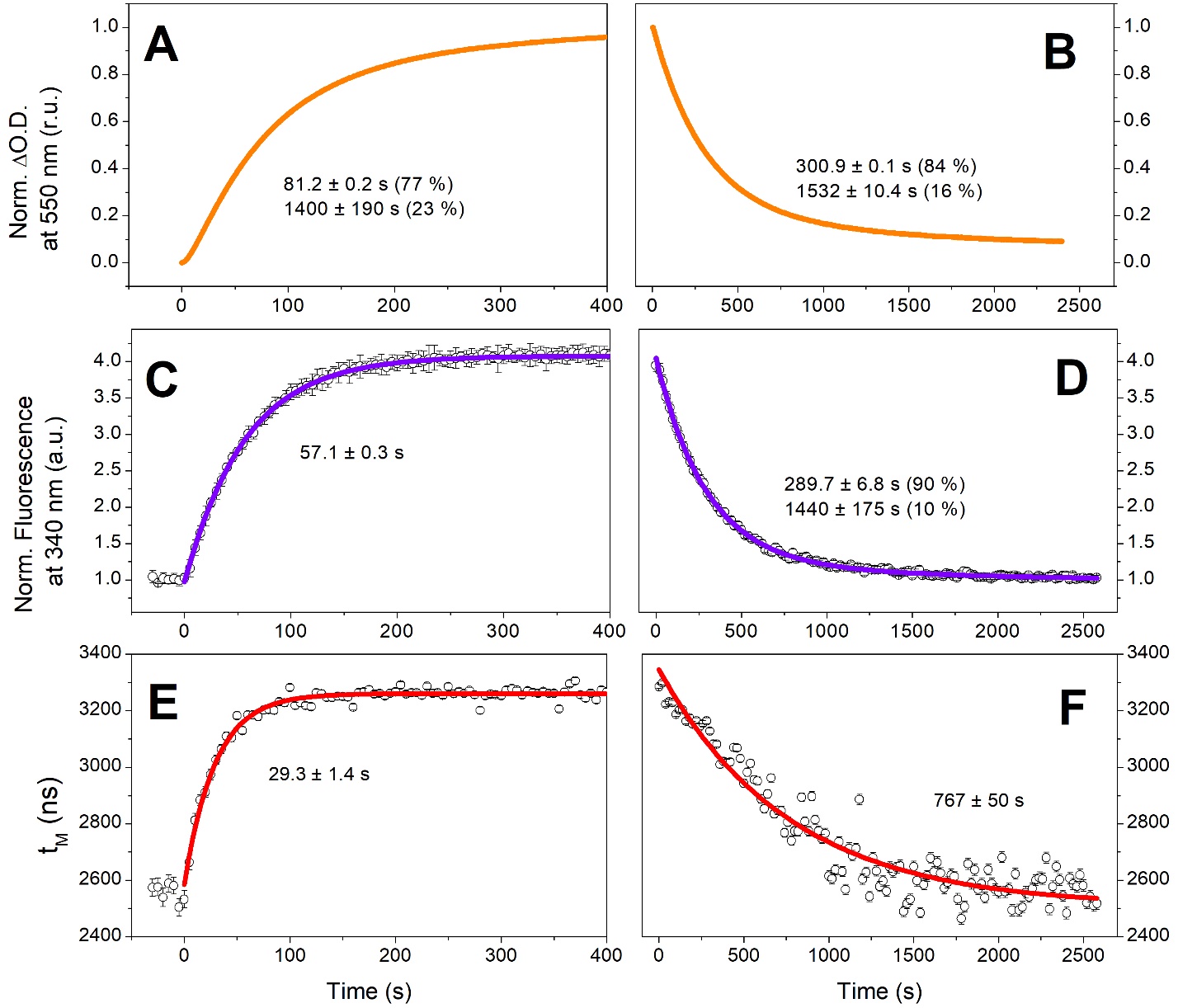


**Figure S4.** Temporal components of the photocycle of the OCP-3FH protein. Left column - photoactivation of OCP-3FH by a 445 nm LED of 5 mW power, right column – subsequent relaxation after the end of illumination. (**A**, **B**) – changes of optical density at 550 nm. (**C**, **D**) – changes of Trp-288 fluorescence intensity at 340 nm. (**E**, **F**) – changes of the average fluorescence lifetime of Trp-288. Experiments were conducted at 0 °C. Each data point represents the average of four independent experiments. In order to obtain panels C-F, 16,640 fluorescent decay curves were processed. Time-courses were approximated by a sum of decaying exponential functions. Numbers represent characteristic time constants and standard deviations.
